# Richter Transformation of Chronic Lymphocytic Leukaemia: Biology, Diagnosis, and an Evolving Therapeutic Landscape

**DOI:** 10.3390/biomedicines14102283

**Published:** 2026-10-08

**Authors:** Juan Marquet-Palomanes, Fernando Martin-Moro, Jose A. Garcia-Vela

**Affiliations:** 1 Department of Haematology, Ramón y Cajal University Hospital/Ramón y Cajal Health Research Institute (IRYCIS), 28034 Madrid, Spain; fmartinmoro@usal.es; 2 Doctoral Programme in Health Sciences, University of Alcala, 28805 Alcala de Henares, Spain; 3 Department of Haematology, Puerta de Hierro University Hospital, 28222 Majadahonda, Spain; garciavela.joseantonio@gmail.com

**Keywords:** Richter transformation, chronic lymphocytic leukaemia, diffuse large B-cell lymphoma, Hodgkin lymphoma, bispecific antibodies, BTK inhibitors, CAR T-cell therapy

## Abstract

Richter transformation (RT) is the development of an aggressive lymphoma in a patient with a previous or concomitant diagnosis of chronic lymphocytic leukaemia (CLL)/small lymphocytic lymphoma (SLL). Most cases are of the diffuse large B-cell lymphoma (DLBCL) type; the Hodgkin lymphoma variant and other lymphoma subtypes are less frequent. RT affects 2–10% of patients with CLL, and survival has historically been measured in months. Approximately 80% of DLBCL-RT cases are clonally related to the underlying CLL and are enriched for unmutated IGHV and high-risk genetic lesions such as *TP53* disruption, *NOTCH1* mutation, *CDKN2A/B* loss and *MYC* activation, and they are associated with poorer outcomes than the minority of clonally unrelated cases, which behave as de novo DLBCL. Multi-omic and single-cell studies indicate that the transformed clone is seeded early. Chemoimmunotherapy produces short-lived responses, and treatment has moved towards targeted and immune-based options including BTK inhibitors, CD20 × CD3 bispecific antibodies, checkpoint-inhibitor combinations and CD19 CAR T-cell therapy, with allogeneic transplantation used as consolidation in fit responders. This review summarises the current data on biology, diagnosis, prognosis and treatment of RT and proposes a practical approach.

## 1. Introduction

A century ago, Maurice Richter described a rapidly fatal “reticular cell sarcoma” in a patient with lymphatic leukaemia [1]. The transformation of chronic lymphocytic leukaemia (CLL)/small lymphocytic lymphoma (SLL) into an aggressive lymphoma remains one of the main challenges in the field of lymphoid tumours. Richter transformation (RT) usually manifests as diffuse large B-cell lymphoma (DLBCL), which accounts for 90–95% of cases. Transformation to Hodgkin lymphoma and other histologies are infrequent and are also recognised in the latest World Health Organisation classification [2,3,4].

Despite the advances in CLL care brought by BTK and *BCL2* inhibitors, RT has not benefited to the same degree. DLBCL-RT is characterised by rapid clinical progression, poor responses to chemoimmunotherapy and short survival [5,6]. Its incidence has stayed broadly similar across the chemoimmunotherapy and novel-agent eras. The lack of advances has driven two parallel efforts: a deeper analysis of the biology that separates RT from both its parent CLL clone and de novo DLBCL, and the development of targeted and immune-based therapies. In 2025 the European Research Initiative in CLL (ERIC) published the first international consensus statement on the diagnosis, evaluation and research of RT, offering a common framework for practice and trial design [7]. This review summarises current evidence on the biology, diagnosis, prognosis and treatment of the disease. To prepare this narrative review, we searched PubMed/MEDLINE for English-language articles published between January 2000 and August 2026, combining the terms “Richter transformation” or “Richter syndrome” with “chronic lymphocytic leukaemia” and terms related to pathogenesis, diagnosis, prognosis and treatment. Ongoing trials were identified through ClinicalTrials.gov, and the reference lists of retrieved articles and current guidelines were hand-searched. No formal quality assessment was performed.

## 2. Epidemiology and Risk Factors

RT develops in 2–10% of patients with CLL, at a rate of 0.5–1% per year. Population-based cohorts show that transformation, although uncommon, remains a persistent risk throughout the disease course [8]. The risk concentrates in patients whose CLL carries high-risk biological features. Unmutated *IGHV* status, *TP53* disruption, *NOTCH1* mutation, stereotyped B-cell receptors (particularly subset #8) and a complex karyotype have all been linked to a higher probability of transformation [9,10,11]. Some immunogenetic contexts are especially informative: subset #8 with mutated *NOTCH1*—whose mutation favours its constant signalling and transcription of oncogenes—, which favours autoreactive B-cell receptors, carries one of the highest transformation risks reported [12,13].

### Impact of CLL Therapy: Focus on Directed Treatment

Whether CLL treatment increases the risk of transformation in the targeted-therapy era has been debated. The high RT rates (up to 25%) seen in the first trials of Bruton tyrosine kinase inhibitors (BTKi) and B-cell lymphoma 2 inhibitors (BCL2is) in relapsed disease probably reflected heavily pre-treated and high-risk populations rather than a transforming effect of the drugs [6,14]. A large single-institution analysis found no rise in RT incidence after targeted agents were introduced, with a 5-year rate from the first treatment of 3.7% before targeted therapy and 2.2% during the targeted-therapy era. In a time-dependent analysis the excess risk came from chemoimmunotherapy (hazard ratio, 4.2, for CIT alone versus no prior treatment), whereas targeted agents alone conferred no added risk (hazard ratio 1.2) [6]. Moreover, transformation is not restricted to treated patients: in the real-world series of the Spanish CLL Study Group (GELLC), 28% of patients had not received any CLL-directed therapy at the time of RT diagnosis [15]. Transformation still occurs during continuous BTK inhibition, but it seems related to baseline biology. In one large ibrutinib-treated cohort (N = 976), DLBCL-RT occurred in 8.5% of patients (7-year incidence 15.6%); it was independently predicted by age <70 years and *TP53* disruption, with a trend for prior treatment, and the 7-year rate rose from 4% in patients with zero or one of these three factors to 22.6% in those with all three [16]. In a separate series, progression on ibrutinib, elevated LDH and progressive lymphadenopathy without lymphocytosis identified patients in whom biopsy to exclude RT is warranted [17].

## 3. Pathobiology and Molecular Landscape

### 3.1. Clonal Relatedness

The key biological and prognostic question in RT is whether the aggressive lymphoma is clonally related to the original CLL. Comparing the immunoglobulin heavy-chain (*IGHV-D-J*) rearrangement of the CLL and the aggressive lymphoma establishes clonal identity [18]. Approximately 80% of DLBCL-RT cases are clonally related to the CLL, often show an activated B-cell-like phenotype (ABC) and represent a true transformation through linear or branched clonal evolution. The remaining ~20% are clonally unrelated and share immune and genomic features with de novo DLBCL. The distinction is crucial for the outcome. Clonally related RT carries a poor prognosis, with a median survival of 8–16 months, whereas clonally unrelated cases may survive several years, close to de novo DLBCL [19,20,21]. Because *IGHV* sequencing is not universally available and can be technically demanding, affordable surrogate markers have been proposed. PD-1 is expressed by the large-cell component in most DLBCL-RT, and its presence correlates with clonal relatedness. However, PD-1 evaluation by immunochemistry should not replace molecular assessment of clonal relatedness [22]. An immune-evasion phenotype, with frequent PD-1/PD-L1 axis upregulation, is characteristic of DLBCL-RT and separates it from de novo DLBCL, which also supports checkpoint-inhibitor-based treatment [23]. In a phenotypic study of 63 DLBCL-RT cases, the transformed lymphoma partly retained the immunophenotype of the antecedent CLL. De novo DLBCLs that are diagnosed together with CLL-like monoclonal B-cell lymphocytosis (MBL) shows a similar phenotype and are frequently clonally related to the MBL. This overlap blurs the line between such cases and true RT, suggesting that some of them may also represent transformation [24].

### 3.2. Recurrent Genetic Lesions

Clonally related RT shows significant genomic instability, with more mutations, copy-number alterations and structural rearrangements than CLL [25,26]. The recurrent oncogenic drivers concur on a few pathways: inactivation of the TP53 tumour suppressor gene in 50–60% of cases; activation of NOTCH1 signalling; loss of the CDKN2A/B cell-cycle checkpoint; and MYC activation through translocation, amplification, promoter mutation or loss of MGA, a negative regulator of MYC signalling. At least one of these lesions is present in 90% of patients [27]. MYC activity depends not only on translocation, amplification or promoter mutation but also on post-translational regulation of protein stability, a level of control that remains largely unexplored in RT and deserves further study. Two main genetic routes have been described, one driven by *TP53* disruption and *MYC* activation and the other by *NOTCH1* mutation [27,28]. Recent research based on genomic and transcriptomic profiling has refined this picture, showing deregulation of the DNA-damage response, MAPK pathway, chromatin-modifying and NF-κB programmes, together with RT-specific lesions such as *IRF2BP2*. Beyond recurrent genetic lesions, non-genetic mechanisms are likely to contribute to sustained oncogenic signalling in RT.

B-cell receptor biology is key to RT. About half of clonally related DLBCL-RT display stereotyped immunoglobulin rearrangements that drive chronic, antigen-independent BCR activation, and *NOTCH1* lesions further sustain oncogenic BCR and NF-κB signalling. Murine and in vivo models of CLL-to-RT transformation reproduce convergent evolutionary paths and therapeutic vulnerabilities, and support BCR-directed therapy [27].

### 3.3. Early Seeding of the Disease

Integrated whole-genome, epigenomic and single-cell sequencing have shown that clonally related RT often originates from small, transformation-primed subclones. These can be detected years, sometimes up to two decades, before conventional transformation, at cancer-cell fractions below 1%. RT cells display a transcriptional profile of high oxidative phosphorylation (OXPHOS) and low BCR signalling, and small subclones with this profile can already be detected at CLL diagnosis, years before clinical transformation. Rather than inducing this metabolic shift, BTK inhibitors are thought to suppress the BCR-dependent CLL population while sparing these pre-existing BCR-independent cells, which rely on mitochondrial metabolism and may expand under selective pressure, providing a rationale for the limited activity of single-agent BTK inhibition in RT [29]. These subclones already carry early driver lesions such as *TP53*, *CDKN2A/B* and *NOTCH1* alterations, and they gradually gain the structural and transcriptomic features of RT before clinical emergence. Reconstruction of the evolutionary history confirms that divergent *TP53*-mutated subclones can coexist for years before one gains a selective advantage. RT is therefore better viewed as an early-established trajectory than as a late event, which raises the possibility that ultrasensitive detection could identify patients who might develop RT in the future [29,30]. In this context, liquid biopsy is an attractive tool. Because RT usually arises in nodal or extranodal sites and may be spatially heterogeneous, analysis of plasma cell-free DNA (cfDNA) can capture transformation-specific alterations that are not represented in circulating CLL cells [30].

## 4. Clinical Presentation and Diagnosis

RT should be suspected when a patient with CLL develops rapidly enlarging, often asymmetric lymphadenopathy, B symptoms (non-infectious fever, night sweats, weight loss), a sudden rise in serum lactate dehydrogenase (LDH), hypercalcaemia, extranodal disease, or clinical deterioration out of proportion to the underlying CLL [5]. Because these features can mimic CLL progression and no single sign is specific enough, histological confirmation is always required [7].

The ERIC consensus recommends an excisional or generous core-needle biopsy of the most metabolically active site [7]. ^18^F-fluorodeoxyglucose positron emission tomography/computed tomography (^18^F-FDG PET/CT) helps guide this step: a high maximum standardised uptake value (SUVmax) ≥ 10 increases the likelihood of transformation and should direct the biopsy to the most avid lesion. SUVmax < 5 has a high negative predictive value for RT but does not exclude RT when clinical suspicion is high. [2]. The histological hallmark of DLBCL-RT is confluent sheets of large, transformed B-cells that efface nodal architecture; an expert haematopathologist should establish the diagnosis in accordance with current classifications and guidelines [2]. It has been reported that DLBCL-RT cases somehow retain the CLL phenotype, showing variable expression of markers frequently found in CLL such as CD43, CD5, CD200, and CD23. One study suggested that the DLBCL-RT phenotype may be modulated by factors related to the preceding CLL, such as prior therapy exposure, and that the CLL-like phenotypic profile observed in DLBCL-RT patients is not found in de novo DLBCL cases [24,31]. Determination of clonal relatedness by *IGHV* sequencing, together with *TP53* status, is strongly recommended, and the ERIC statement sets out how these evaluations should be performed and reported [7,32,33].

Pseudo-Richter transformation is a diagnostic pitfall. Abrupt interruption of BTKi therapy can trigger a disease flare with rapidly enlarging, FDG-avid lymphadenopathy, B-symptoms and rising LDH levels, and the biopsy may show sheets of large cells with a high Ki-67 proliferation index that resemble RT. The infiltrate retains the CLL immunophenotype, the flare typically appears within days of stopping the drug, and it resolves once the BTKi is resumed [34].

## 5. Prognostic Assessment

Several factors define the prognosis of RT. Beyond clonal relatedness and *TP53* status, adverse clinical features include poor performance status, elevated LDH, thrombocytopenia, bulky disease, prior CLL-directed therapy, complex karyotype and advanced stage [2,5]. Previous exposure to targeted agents is a particularly poor prognostic sign. RT arising in patients already treated with, and especially double refractory to, BTK and BCL2 inhibitors carries a dismal outcome, with median overall survival under one year (around 8 months among patients transforming on ibrutinib). By contrast, RT in treatment-naïve, chemoimmunotherapy-unexposed patients approaches the outcome of de novo DLBCL [7,14,35,36]. The Tsimberidou (“RS”) score combines five clinical variables (ECOG performance status, LDH, platelet count, tumour size and number of prior therapies) to stratify survival into risk groups. In the Mayo Clinic cohort of 204 patients, prior CLL treatment and elevated LDH were associated with inferior survival. TP53 disruption was associated with shorter survival in univariable analysis but not in the multivariable model; clonal relatedness was assessed in a limited subset [4]. These tools were developed largely in the chemoimmunotherapy era and need re-evaluation as novel agents change outcomes [37].

In practical terms, two opposite scenarios behave almost as different diseases: treatment-naïve, clonally unrelated, *TP53*-wild-type RT, and heavily pre-treated, clonally related, *TP53*-disrupted RT. Each scenario should be counselled and treated accordingly [19,35].

## 6. Treatment

**Figure 1:**
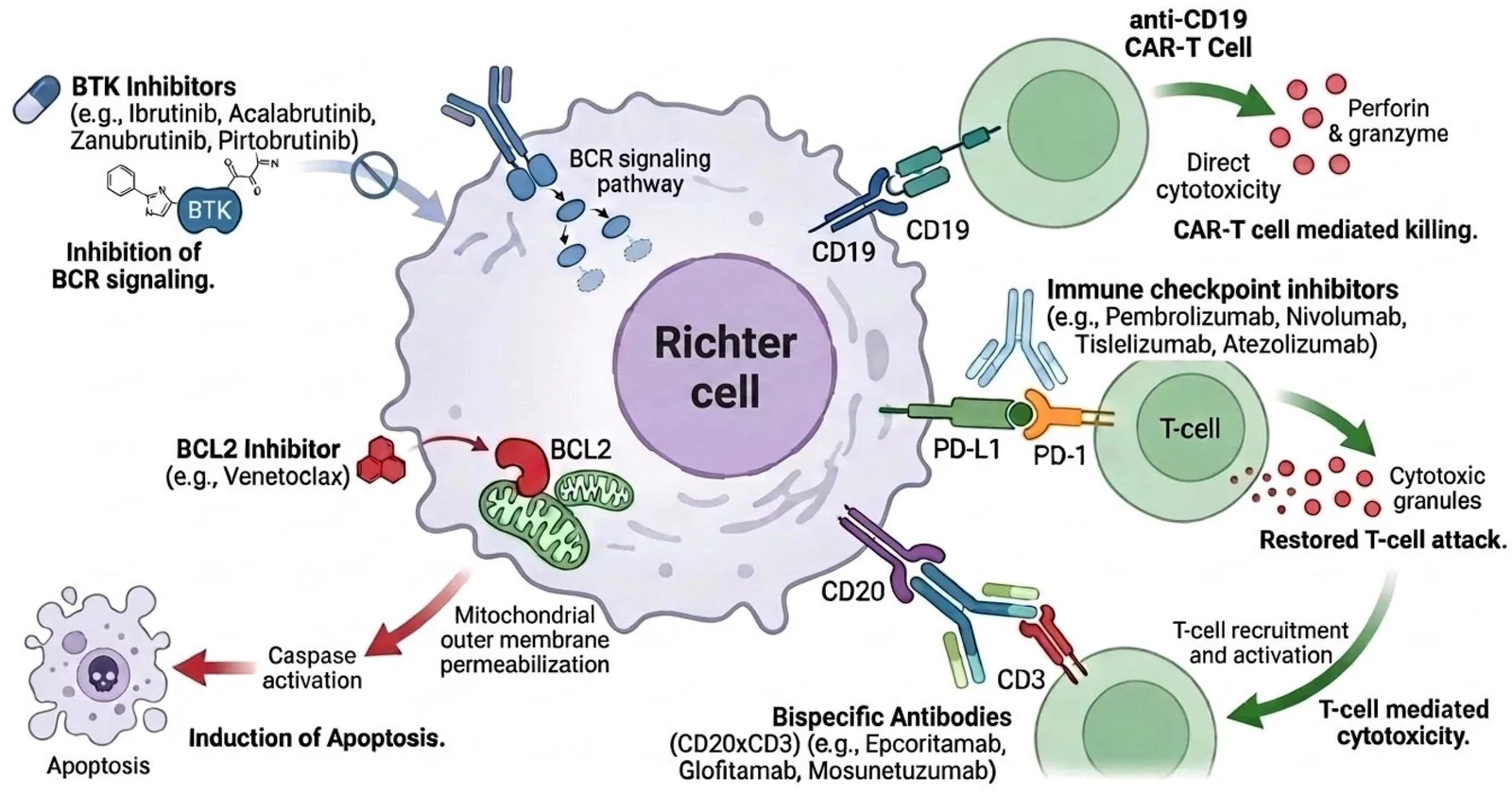
Mechanisms of action of novel therapeutic approaches in RT. BTKis block BCR signalling. BCL2 inhibitors trigger mitochondrial outer membrane permeabilisation, caspase activation and apoptosis. PD-1 or PD-L1 inhibitors restore T-cell-mediated cytotoxicity. CD20 × CD3 bispecific antibodies bring T cells into contact with the tumour cell, inducing T-cell activation and tumour cell killing. CAR T cells recognise CD19 and exert direct cytotoxicity through perforin and granzyme release.

No therapy is approved specifically for RT, and a clinical trial is the preferred option whenever available. Two questions guide the strategy: is the disease clonally related and *TP53*-disrupted, and is the patient fit enough for cellular therapy or transplantation? Indeed, fitness is a real limiting factor: a substantial proportion of patients—up to one quarter in some real-world series—receive only supportive or palliative care due to frailty, comorbidities or poor performance status, in clear contrast to de novo DLBCL [15]. The subsections below summarise the main new treatment modalities and emerging strategies, and representative trials are shown in Table 1. The 2026 European Hematology Association (EHA) guidelines on CLL now include a dedicated section on Richter transformation. They encourage clinical-trial enrolment and, for eligible patients, novel-agent strategies (bispecific antibodies, CAR T-cell therapy and BTK degraders), with allogeneic transplantation as consolidation, in line with the options discussed here [38]. New treatment approaches are summarised in Figure 1.

### 6.1. Chemoimmunotherapy

Anthracycline-based chemoimmunotherapy has been the standard approach for RT. R-CHOP yields complete response rates of only about 20–30% and a median overall survival of 6–12 months, well below the results of the same regimen in de novo DLBCL [6,37]. Intensified or platinum-based regimens, including R-EPOCH and R-hyper-CVAD, offer modest gains at the cost of greater toxicity [39,40]. In a large R-EPOCH cohort, a complex CLL karyotype identified a group with markedly inferior progression-free and overall survival [40]. Chemoimmunotherapy is best used either as a bridge to cellular therapy in fit patients or as part of the novel-agent strategies described below.

### 6.2. Bruton Tyrosine Kinase Inhibitors

Given the central role of BCR signalling in RT, BTK inhibition has been proposed to improve outcomes. Covalent BTK inhibitors produce short-lived responses, and their activity is reduced in patients previously treated with these agents. Ibrutinib monotherapy has not been tested in a dedicated RT trial; the evidence comes from small retrospective series showing brief responses in heavily pre-treated patients [41]. Acalabrutinib is a covalent BTK inhibitor assessed prospectively as monotherapy in RT: in the phase 1–2 ACE-CL-001 Richter cohort (N = 25) it produced an overall response rate (ORR) of 40% (8% CR), a median duration of response of 6.2 months and a median progression-free survival (PFS) of 3.2 months [42]. The most encouraging data come from the non-covalent inhibitor pirtobrutinib, which inhibits both wild-type and C481-mutant BTK. In the RT cohort of the BRUIN study (N = 82, mostly heavily pre-treated, including prior covalent BTK-inhibitor exposure), pirtobrutinib monotherapy achieved an ORR of 50% with 13% complete responses (CR), a median duration of response of 7.4 months and a median overall survival (OS) of 12.5 months [43]. Several responders were successfully bridged to cellular therapy, although durable remissions with single-agent pirtobrutinib remain uncommon. Adding a covalent BTK inhibitor to chemoimmunotherapy is being tested in the randomised phase II STELLAR trial, which evaluates the acalabrutinib added to the R-CHOP backbone [44]. BTK degraders represent a further investigational approach. These agents promote targeted proteasomal degradation of BTK rather than directly inhibiting its kinase activity, potentially overcoming selected mechanisms of resistance to BTK inhibitors. In a mechanistically distinct setting, heat stress-induced degradation of BCR::ABL1 has been demonstrated in Philadelphia chromosome-positive acute lymphoblastic leukaemia [45]. This finding illustrates the broader therapeutic potential of oncoprotein degradation, although it does not establish the efficacy of pharmacological BTK degraders in RT. Early phase 1 data with BGB-16673 in relapsed/refractory RT (CaDAnCe-101, N = 24) showed an ORR of 45.8% although these findings are preliminary.

### 6.3. Bispecific Antibodies

CD20 × CD3 T-cell–engaging bispecific antibodies are among the most active new options in RT. Glofitamab (a 2:1 CD20:CD3 format given after obinutuzumab pre-treatment) produced deep and durable responses in a small phase 1 Richter cohort of 11 patients. Median PFS was only 3.5 months, reflecting a subset with no response, but the complete responses were long-lasting and persisted for 55–74 months in individual responders without further consolidation [46]. Epcoritamab, a subcutaneous CD20 × CD3 bispecific antibody, was evaluated in the dedicated EPCORE CLL-1 trial. In the monotherapy cohort (arm 2A, N = 42; 48% with *TP53* aberration), the ORR was 48% and the CR rate was 40% at a median follow-up of 22.9 months. Results were better when epcoritamab was used as first-line RT-directed therapy (N = 21) with an ORR of 57%, CR rate of 52%, and median OS of 27.5 months [47]. Mosunetuzumab, an intravenous CD20 × CD3 bispecific antibody, was tested as monotherapy in a phase I/II cohort of 20 patients with relapsed/refractory DLBCL-type RT. It produced an ORR of 40% and a CR rate of 20%, with a median PFS of 3.4 months and a median OS of 10.2 months, confirming class activity in a heavily pre-treated population [48]. Early combination cohorts (epcoritamab with lenalidomide or with R-CHOP) have reported higher response rates and are still under investigation, and a Spanish multicentre phase II trial (GELLC-9-Richter, NCT06521996) is evaluating mosunetuzumab plus CHOP as first-line therapy in previously untreated RT. Cytokine release syndrome is the characteristic toxicity, but it is mostly low-grade and is mitigated by step-up dosing and corticosteroid prophylaxis [46,47,48].

### 6.4. Checkpoint-Inhibitor-Based Combinations

The immunosuppressive environment of RT, with frequent PD-1/PD-L1 axis upregulation and a recurrent immune-evasion phenotype, provides a rationale for checkpoint blockade [21,22]. Single-agent activity is limited and largely confined to the transformed compartment. Pembrolizumab produced responses mainly in RT rather than in relapsed CLL (ORR 44% versus 0%), including in patients progressing after ibrutinib and with higher PD-L1 expression [49]. Combinations appear more effective, although the evidence is heterogeneous. An early phase 1/2a study of ibrutinib plus nivolumab reported an ORR of 65% in the RT cohort [50], but a dedicated phase 2 study was less favourable: nivolumab plus ibrutinib achieved a 42% response rate with a median OS of 13 months and little benefit beyond ibrutinib alone [51]. PD-1 blockade with PI3K inhibition has shown modest activity in heavily pre-treated disease [52]. The recent combination trials are more promising. The phase 2 RT1 trial paired the PD-1 inhibitor tislelizumab with the BTK inhibitor zanubrutinib and reported an ORR of 58.3% (18.8% CRs) and a median PFS of 10 months, with good tolerability and few immune-related events [53]. The MOLTO combination of atezolizumab, venetoclax and obinutuzumab produced high responses (ORR 67.9%; 28.6% of them CR), although durability was limited (12-month PFS 42.9%) [54]. Taken together, these data support checkpoint/BTK-inhibitor and checkpoint/venetoclax combinations as active and reasonably tolerated options, especially for patients unsuitable for chemotherapy.

### 6.5. Venetoclax-Based Approaches

Venetoclax has been combined with chemoimmunotherapy and with other targeted agents in RT. Single-agent activity is modest and rarely durable, and transformation can emerge during venetoclax therapy for CLL [55]. Venetoclax with dose-adjusted R-EPOCH (VR-EPOCH) showed encouraging results in a phase 2 trial. Among 26 treated patients the ORR was 62%, and the complete response rate was 50%, rising to 80% and 65% respectively among the 20 patients who received at least one VR-EPOCH cycle, with a median PFS of 10.1 months and a median OS of 19.6 months [56]. A multicentre study of 62 patients mapped the activity of venetoclax-based combinations across regimens. Venetoclax plus R-CHOP produced the best outcomes (ORR 54%, complete response 46%, median OS not reached), whereas venetoclax plus a BTK inhibitor or plus other intensive chemoimmunotherapy gave lower response rates (36% and 52%) and shorter survival (median OS of 14.3 and 9.0 months) [57]. Chemotherapy-free doublets are also under study: a phase 1/2 trial of duvelisib plus venetoclax included a small Richter cohort (N= 9), four of whom responded, including three who achieved CR [58].

### 6.6. CD19-Directed CAR T-Cell Therapy

CD19-directed CAR T-cell therapy offers the prospect of durable remission in a disease otherwise dominated by relapse. Beyond early single-centre activity with axicabtagene ciloleucel [59], several multicentre series now define its role. An international retrospective study (N = 69) reported a 63% overall and 46% complete response rate. Median PFS and OS were short (4.7 and 8.5 months), but the complete responses were durable, with a median duration of 27.6 months [60]. The ERIC study (N = 54) was concordant: an ORR of 65%, complete response in about 50%, median PFS of 31.6 months among complete responders and median OS of around 14 months [61]. The largest real-world dataset, a CIBMTR registry of 140 patients, showed 2-year progression-free and overall survival of 32.5% and 46.6%, with a low non-relapse mortality of 8.7% [62]. Academic point-of-care manufacturing is feasible: the Spanish ARI-0001 (varnimcabtagene autoleucel) product produced responses in a small CLL/Richter cohort. Among five treated Richter patients, three remained disease-free for up to 26.7 months, with mostly low-grade cytokine release syndrome and no neurotoxicity, although CD19-negative relapses showed antigen escape [63]. Across these series, cytokine release syndrome is common but mostly low-grade, whereas immune effector cell-associated neurotoxicity is more prominent (grade ≥ 3 up to about 20–37% in the larger cohorts) and needs experienced management. Overall, CD19-directed CAR T-cell therapy can secure durable remissions, and possibly cure, in a subset of patients with RT. Outcomes remain inferior to de novo large B-cell lymphoma, and early response has emerged as a key determinant of long-term benefit [60,61].

### 6.7. Allogeneic Haematopoietic Stem-Cell Transplantation

Allogeneic haematopoietic stem-cell transplantation (allo-HSCT) is still the only conventionally established potentially curative option, and it is best positioned as consolidation in fit patients who have responded to induction. Retrospective series from the European Society for Blood and Marrow Transplantation and single centers report three-year OS of about 40–50%, with the best results in patients transplanted in complete or partial remission [64,65,66]. Its use is limited by patient age, comorbidity and treatment-related mortality. Novel agents (pirtobrutinib, bispecific antibodies or CAR T-cell therapy) are increasingly used to achieve a deep remission that can then be consolidated with allo-HSCT in selected, transplant-eligible patients [43,61].

**Table 1:** Selected prospective and multicentre studies of novel agents in DLBCL Richter transformation.

Agent/Regimen	Study (Design)	N (RT)	ORR/CR	Key Outcome
Pirtobrutinib	BRUIN (phase 1/2 subgroup) [43]	82	50%/13%	mDOR 7.4 mo; bridge to allo-HSCT
Epcoritamab (1L)	EPCORE CLL-1 arm 2A (ph 1b/2) [47]	21	57%/52%	Median OS 27.5 mo
Epcoritamab (all)	EPCORE CLL-1 arm 2A (ph 1b/2) [47]	42	48%/40%	Median OS 13.0 mo
Glofitamab	Phase 1 (single-agent) [46]	~11	64%/46%	Durable CRs (55–74 mo); mPFS 3.5 mo
Mosunetuzumab	Phase I/II (single-agent) [48]	20	40%/20%	mPFS 3.4 mo; mOS 10.2 mo
Tislelizumab + zanubrutinib	RT1 (phase 2) [53]	48 (of 59)	58%/19%	Median PFS 10.0 mo
Nivolumab + ibrutinib	Phase 2 (MDACC) [51]	24	42%/—	mDOR ~15 mo (responders)
Venetoclax + R-EPOCH	VR-EPOCH (phase 2) [56]	26	62%/50%	mOS 19.6 mo; CR 65% in VR-EPOCH
Atezolizumab + venetoclax + obinutuzumab	MOLTO (phase 2) [54]	28	68%/36%	12-mo PFS/OS 43%/64%
CD19 CAR T-cell	ERIC (retrospective) [61]	54	65%/46–50%	mOS 14.4 mo; mPFS 31.6 mo in CR
CD19 CAR T-cell	CIBMTR (registry) [62]	140	—/—	Largest series; 2-y PFS/OS 32.5%/46.6%; NRM 8.7%
R-CHOP (historical)	Retrospective cohorts [6]	—	~20–30% CR	Median OS ~6–12 mo

1L, first line; allo-HSCT, allogeneic haematopoietic stem-cell transplantation; CR, complete response; DOR, duration of response; ERIC, European Research Initiative on CLL; mDOR, median DOR; ORR, overall response rate; OS, overall survival; PFS, progression-free survival; RT, Richter transformation. Figures are from the cited primary reports; cross-trial comparison is limited by differences in populations and follow-up.

## 7. Hodgkin Lymphoma Variant of Richter Transformation

The Hodgkin lymphoma variant (HvRT) is a rare and distinct form of transformation. It affects about 1% of patients with CLL and is diagnosed 4–6 years after the CLL diagnosis, mostly in elderly men, often of mixed-cellularity subtype [4,67,68]. Two patterns have been described: a “Hodgkin-like” lesion, in which Reed–Sternberg (HRS) cells appear within an unmodified CLL background, and true classic Hodgkin lymphoma transformation, defined by diagnostic HRS cells in a polymorphous inflammatory environment. Rather than discrete entities, these probably form a biological continuum [69]. HRS cells are Epstein–Barr-virus positive in about 70% of cases and may be clonally related or unrelated to the CLL, so demonstrating clonal identity carries less prognostic value than in DLBCL-RT [67]. Outcomes are better than in DLBCL-RT (median survival about 39–44 months vs. 9 months) but worse than in de novo Hodgkin lymphoma, with advanced age, rather than histological pattern, as the main adverse factor [67,68]. In the largest population-based series, two-year PFS and OS were 47% and 57% [70]. Prior CLL-directed therapy does not appear to worsen outcome, and patients are managed with Hodgkin-directed regimens rather than DLBCL-RT strategies [70]. Brentuximab-vedotin + AVD has shown activity in a small case series of HvRT, but evidence remains insufficient to define its comparative efficacy or tolerability in this population [71].

## 8. A Practical, Evidence-Based Approach

**Figure 2:**
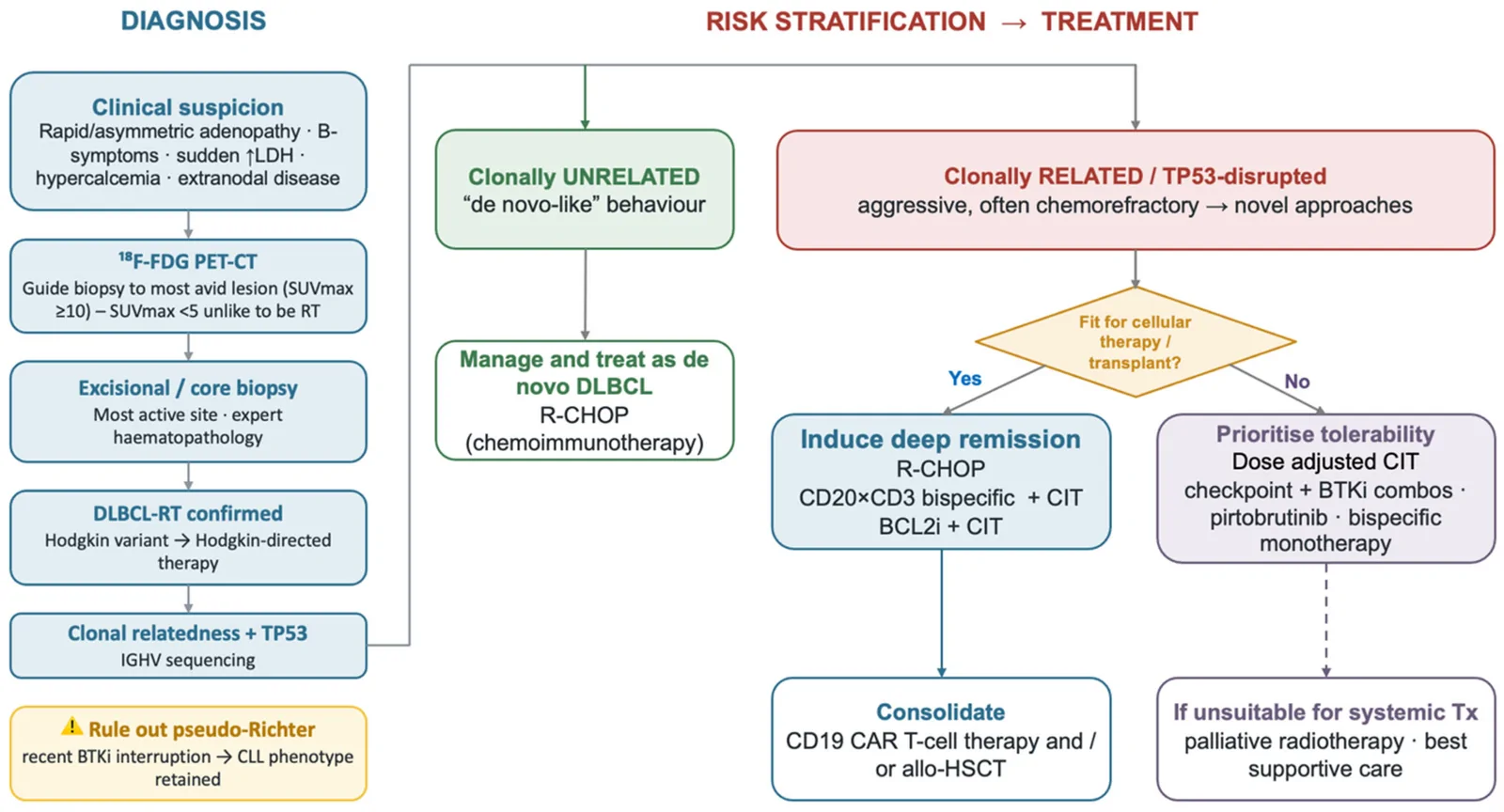
Diagnostic and therapeutic approach for RT (the algorithm reflects the authors’ opinion). Fitness for cellular therapy is assessed individually, as no validated criteria exist for RT. Patients are generally considered fit if they have an Eastern Cooperative Oncology Group (ECOG) performance status of 0–1 (selected patients with ECOG 2 attributable to the disease), adequate cardiac, renal, hepatic and pulmonary function. Dashed arrow: transition to palliative/supportive care based on clinical judgment.

In the absence of randomised head-to-head data, management should be based on clonal relatedness and *TP53* status [2,38,72,73]. A schematic approach is provided in Figure 2, but a few points are worth emphasising:

- Confirm the diagnosis rigorously. Biopsy the most FDG-avid lesion (SUVmax ≥ 10), obtain expert pathology review, and determine clonal relatedness and *TP53* status.
- Stratify by clonal relatedness. Clonally unrelated, de novo-like RT can be treated as DLBCL; clonally related, *TP53*-disrupted RT is an aggressive, often chemorefractory disease that needs the novel approaches described above.
- Induce a deep remission. In transplant- or CAR-T-eligible patients, favour regimens with high complete-response potential if available (bispecific antibodies such as epcoritamab, glofitamab or mosunetuzumab or novel-agent combinations) to enable consolidation and durable remission.
- Choose the cellular therapy deliberately in patients eligible for both. Without randomised comparisons, CAR T-cell therapy is a reasonable first option given its favourable toxicity profile and because it does not preclude transplantation, reserving allo-HSCT for consolidation in selected cases or as salvage after CAR-T failure.
- Prioritise tolerability in unfit patients. For those unsuitable for intensive therapy, checkpoint/BTK-inhibitor combinations, single-agent pirtobrutinib or bispecific monotherapy provide meaningful, better-tolerated disease control. Patients who cannot receive systemic treatment may benefit from palliative radiotherapy and best supportive care.

## 9. Future Directions

Several lines of work will shape RT research. Because transformation is seeded early [28,29], one priority is the search for ultrasensitive measurable residual disease and liquid-biopsy tools that can identify pre-transformation subclones and perhaps allow pre-emptive treatment. New agents continue to broaden the therapeutic landscape: BTK degraders, which retain activity against BTK-inhibitor-resistant clones, have shown encouraging early activity in RT, and their rational combination and sequencing with bispecific antibodies, non-covalent BTK inhibitors, venetoclax and CAR T-cell therapy may produce durable remissions and, in some patients, a realistic prospect of cure [43,47,61]. Harmonised diagnostic and response criteria under the ERIC consensus should improve the comparability and quality of upcoming trials [7]. A better understanding of metabolic reprogramming, the immunosuppressive microenvironment and evolutionary vulnerabilities may reveal new targets [27].

## 10. Conclusions

Richter transformation remains the most feared complication of CLL. Clonal relatedness and *TP53* status continue to determine prognosis, and chemoimmunotherapy alone is no longer an adequate standard for clonally related disease if long-term remission is desired. Non-covalent BTK inhibition, CD20 × CD3 bispecific antibodies (especially when used early), checkpoint-inhibitor combinations and CD19 CAR T-cell therapy have each shown clinically meaningful activity, and allogeneic transplantation retains a consolidative, potentially curative role in fit responders. With the first international consensus now in place and several combination strategies in trials, the main task is to turn increasingly frequent responses into durable, and ultimately curative, remissions.

## Data Availability

No new data were created or analyzed in this study. Data sharing is not applicable to this article.
